# The mutational pattern of homologous recombination (HR)-associated genes and its relevance to the immunotherapeutic response in gastric cancer

**DOI:** 10.20892/j.issn.2095-3941.2020.0089

**Published:** 2020-12-15

**Authors:** Yue Fan, Haifeng Ying, Xueying Wu, Huan Chen, Ying Hu, Henghui Zhang, Lijia Wu, Ying Yang, Beibei Mao, Lan Zheng

**Affiliations:** 1 Department of integrated Traditional Chinese Medicine and Western Medicine, Zhongshan Hospital, Fudan University, Shanghai 200032, China; 2 Department of Traditional Chinese Medicine, Ruijin Hospital, Shanghai Jiaotong University, Shanghai 200025, China; 3 Genecast Precision Medicine Technology Institute, Beijing 100191, China; 4 Center of Integrative Medicine, Beijing Ditan Hospital, Capital Medical University, Beijing 100015, China

**Keywords:** Gastric cancer, homologous recombination deficiency, immunotherapy, biomarker

## Abstract

**Objective:**

Currently, there is an urgent need to identify immunotherapeutic biomarkers to increase the benefit of immune checkpoint inhibitors (ICIs) for patients with gastric cancer (GC). Homologous recombination deficiency (HRD) can modify the tumor immune microenvironment by increasing the presence of tumor-infiltrating lymphocytes and therefore might serve as a biomarker of immunotherapeutic response. We aimed to analyze the mutational pattern of HR-associated genes in Chinese patients with GC and its relevance to the tumor immune profile and clinical immunotherapeutic response.

**Methods:**

A panel of 543 cancer-associated genes was used to analyze genomic profiles in a cohort comprising 484 Chinese patients with GC. Correlations between HR gene mutations and tumor immunity or clinical outcomes were identified *via* bioinformatic analysis using 2 GC genomic datasets (TCGA and MSK-IMPACT).

**Results:**

Fifty-one of the 484 (10.54%) patients carried at least one somatic mutation in an HR gene; *ATM* (16/484, 3.31%) was among the most frequently mutated HR genes in the Chinese cohort. Mutations in HR genes were associated with elevated tumor mutational burden, enhanced immune activity, and microsatellite instability status. In the MSK-IMPACT cohort comprising 49 patients with stomach adenocarcinoma or gastroesophageal junction adenocarcinoma treated with ICIs, patients with HR-mut GC (*n* = 12) had significantly better overall survival than those with HR-wt GC (*n* = 37) (log-rank test, *P* < 0.05).

**Conclusions:**

Our data suggest that detection of somatic mutations in HR genes might aid in identifying patients who might benefit from immune checkpoint blockade therapy.

## Introduction

Gastric cancer (GC) is the third leading cause of cancer death worldwide^1^. The morbidity and mortality of GC are particularly high in East Asia, as is the incidence of various environmental and genetic factors affecting the disease^1,2^. Although the therapeutic effects of surgical resection with adjuvant or neoadjuvant radiotherapy and chemotherapy on early GC are impressive, many patients with GC miss the opportunity to undergo surgical resection because the disease is often not diagnosed until it reaches advanced stages^3^.

Recently, therapeutic strategies based on immune checkpoint inhibitors (ICIs) have been introduced for GC. Unfortunately, in the KEYNOTE-061 (NCT02370498) study, pembrolizumab did not show a significant curative effect in treating patients with advanced gastric or gastroesophageal junction cancer who had a PD-L1 combined positive score of 1 or higher^4^. Accordingly, potential predictive therapeutic biomarkers beyond PD-L1 expression must urgently be identified to help increase the benefit of these therapies in patients.

Numerous studies have demonstrated that homologous recombination deficiency (HRD) can modify the tumor immune microenvironment by increasing the presence of tumor-infiltrating lymphocytes (TILs)^5^ and consequently might serve as a biomarker of immunotherapeutic response. HRD was initially defined by the presence of germline mutations in *BRCA1* and *BRCA2*^6^. However, enabled by the development of next-generation sequencing, several studies have suggested that patients with gynecological cancer with somatic mutations in HR pathway genes are also likely to express the HRD phenotype^7–9^. Nonetheless, the mutational pattern of HR-associated genes and its relevance to the tumor immune profile and clinical immunotherapeutic response in GC remain to be clarified.

The current study first aimed to delineate the mutation rates and characteristics of HR genes in GC in a large Chinese cohort and a cohort from The Cancer Genome Atlas (TCGA). The second aim was to comprehensively assess the correlation between mutated HR genes and tumor immune profiles in the cohort from TCGA. Collectively, our investigation indicated that HR gene mutations are enriched in the Chinese population and that HRD status may predict the response to ICIs in patients with GC.

## Materials and methods

### HR status definition

The HRD phenotype was defined as a nonsilent somatic mutation in any gene in 2 groups (HR-mut group and *BRCA1/2*-mut group). The HR-mut group included patients with at least one nonsilent mutation in any of 10 HR genes: *ATM*^10,11^, *ATR*^12^, *ATRX*^13^, *BRCA1*^14^, *BRCA2*^14^, *CHEK1*^15^, *CHEK2*^16^, *PALB2*^10^, *RAD51C*^17^, or *RAD51D*^17^. Clinical data indicate that *ATR* is a key regulator of cell cycle checkpoints and HR^12^, and that *ATRX* is required for sister chromatid exchange during HR^13^. The *BRCA1/2*-mut group included patients with at least one nonsilent mutation in *BRCA1* or *BRCA2*.

### Patient information and sample collection

To analyze the prevalence of HR gene mutations in patients with GC, we collected genomic data from 925 patients diagnosed with GC from 2 cohorts (Chinese cohort and TCGA cohort). In the Chinese cohort, a total of 502 cases with confirmed GC were screened initially in 2 integrated hospitals (Zhongshan Hospital and Ruijin Hospital), and all patients provided signed informed consent. Briefly, after exclusion of cases without matched blood cells (*n =* 4) and cases failing quality control (*n =* 14), we included 484 cases with GC in the final analysis (**Supplementary Figure S1A**). In the TCGA cohort, a total of 441 cases with both whole exome sequencing and RNA-sequencing data from the TCGA-STAD project were included (**Supplementary Figure S1A**). TCGA single-nucleotide variant (SNV) data were obtained from the GDC Data Portal (https://portal.gdc.cancer.gov/). The inclusion/exclusion criteria for the samples were as follows: 1) only tumor samples were included; 2) the primary site was the stomach; and 3) all silent mutations were ignored. An ICI-treated cohort (MSK-IMPACK cohort) containing 49 patients (with 1 case without matched somatic mutation excluded) with stomach adenocarcinoma (STAD) or gastroesophageal junction adenocarcinoma (GEJ) was used to explore the correlation between HR-mut status and the ICI response. Basic information regarding how the demographics of the Chinese cohort, TCGA cohort, and MSK-IMPACK cohort are derived are shown in **Supplementary Table S1**.

### DNA extraction and sequencing

Formalin-fixed, paraffin-embedded (FFPE) tissue specimens from each patient’s primary tumor were collected for analysis. A blackPREP FFPE DNA Kit (Analytik Jena, Germany) was used to isolate DNA from the FFPE tissue specimens. Whole blood centrifuge (1,600 × g) for 10 min at room temperature was to isolate lymphocytes. A Tiangen Whole Blood DNA Kit (Tiangen, Beijing, China) was used to extract DNA from peripheral blood lymphocytes according to the manufacturer’s instructions. The genomic DNA was fragmented into 150–200-bp segments with a Covaris M220 focused ultrasonicator (Covaris, Massachusetts, USA). A fragmented DNA library was constructed with a KAPA HTP library preparation kit for the Illumina platform (KAPA Biosystems, Massachusetts, USA) according to the manufacturer’s instructions. DNA sequences from a DNA library (NimbleGen SeqCap EZ Library; Roche, Wisconsin, USA) including major tumor-associated genes, were captured with a 543-gene plate and subjected to paired-end sequencing with an Illumina HiSeq X-Ten instrument.

### Variant calling

We used VarScan2 (v2.4.2) to call somatic cell SNVs that matched blood samples. The following parameters were used: 1) number of mutant allele reads > 2; 2) coverage: normal > 50× and tumor > 100×; 3) mutated allele frequency > 2%; 4) nonsynonymous SNVs and 5) indels located in exonic regions; and 6) allele frequency < 0.5% in the database Exome Aggregation Consortum (ExAC).

### Analysis of the tumor mutational burden (TMB) and MSI status in the Chinese cohort

The TMB [mutations per megabase (Mb) DNA] was extrapolated with sequencing data for the gene panel containing 543 cancer-associated genes and was determined by analysis of the number of somatic mutations per Mb. The 75th percentile of the TMB value in this study was selected as the cutoff for defining a tumor as TMB-high (TMB-H).

Tumor sample DNA was sequenced *via* next-generation sequencing with the cancer gene-targeted panel. Seventy target microsatellite loci were examined and compared with those in the genomic data from healthy individuals in the Chinese database. The number of microsatellite loci altered by somatic insertions or deletions was counted for each patient sample. If the unstable locus/passing locus ratio was ≥ 0.3, the microsatellite instability (MSI) status was MSI-high (MSI-H); if the ratio was < 0.3, the MSI status was MSI-low/microsatellite stable (MSI-L/MSS).

Associations among HRD and the TMB, MSI status, neoantigen burden, aneuploidy status, and TIL infiltration in the TCGA cohort

TMB, MSI, immunogenic somatic mutation, copy number variation, and loss of heterozygosity (LOH) data were downloaded from published studies^18–22^ with the cohort from TCGA. The CIBERSORT algorithm reported by Newman et al.^23^ was used to analyze the TIL abundance. Gene expression data collected from TCGA were uploaded to the CIBERSORT web portal (http://cibersort.stanford.edu/), and the algorithm was run with the default base matrix. Our results were analyzed on the basis of the intersection of the genomic data and these downloaded data.

### Immune-related signature analysis

That gene sets for cytolytic activity (granzyme-A and perforin-1), IFN-γ, *etc*, have been used in previous studies^22,24,25^ (**Supplementary Table S3**). TCGA transcriptome profiling data were obtained from GDC Data Portal (https://portal.gdc.cancer.gov/). Expression of each target gene was normalized with transcripts per million (TPM) normalization, and the immune signatures were assessed as the geometric mean of the gene expression level in log_2_(TPM+1).

### Association between HRD and survival outcomes

We compared the overall survival (OS) of patients with HRD (HR-mut or *BRCA1/2*-mut) and non-HRD (HR-wt or *BRCA1/2*-wt) GC in an ICI-treated cohort (MSK-IMPACT cohort)^26^. Kaplan–Meier survival curves were used to illustrate survival differences, and the log-rank test was used to evaluate the significance of survival time differences. We performed survival analyses by using the R programming function “survfit” in the “survival” package.

### Statistical analysis

All statistical analyses were performed in R version 3.6.1 software (Institute for Statistics and Mathematics, Vienna, Austria; www.r-project.org). Fisher’s exact test was applied for comparisons between 2 categorical variables, and the Mann–Whitney *U* test was used for comparisons between 2 continuous variables. Survival analysis was performed with a Kaplan–Meier survival plot, and the log-rank test *P* value was calculated. All differences with *P* < 0.05 were considered statistically significant.

## Results

### Mutational landscape of HR genes in Chinese patients with GC

**Figure 1:**
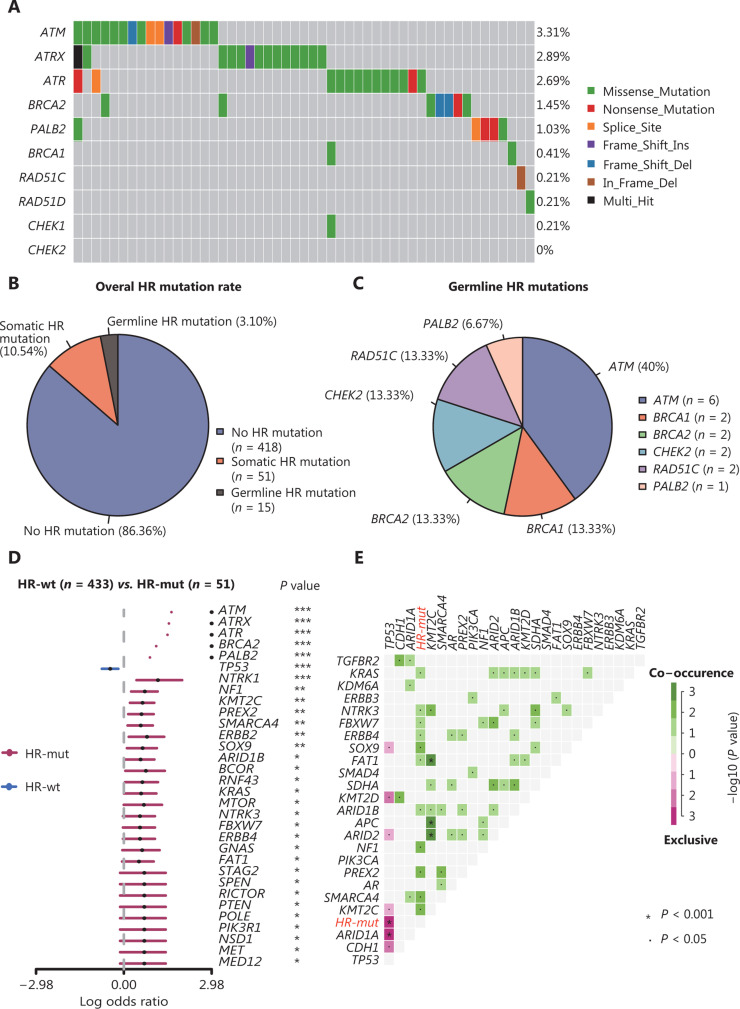
The mutational landscape and genomic patterns of HR genes in GC. (A) Mutational landscape of HR genes in the Chinese cohort. The columns and rows represent patients and genes, respectively, and are sorted in decreasing order by the number of patients in which a gene is mutated. The right panel indicates the frequency of gene mutations. Mutation types are differentiated by colors. (B, C) Gray denotes no mutations. Pie charts illustrating the distribution of HR mutational types (B) and germline mutation frequencies of HR genes (C). (D) Forest plot showing enrichment of mutations in HR-mut and HR-wt by logarithmic odds ratio (*P < 0.05, **P < 0.01, ***P < 0.001). The x-axis is the log odds ratio. (E) Cooccurring and exclusive somatic mutations in the Chinese cohort. *P* values were calculated with Fisher’s exact test. All 63 somatic mutated HR genes were marked as HR-mut. These figures were produced with the “forestPlot” and “somaticInteractions” functions in the maftools package.

Overall, at least one genomic alteration was observed in 93.8% (454/484) of the samples, and the 3 most frequently mutated genes were *TP53* (283/484, 58.47%), *CDH1* (74/484, 15.29%), and *ARID1A* (52/484, 10.74%) (**Supplementary Figure S1A**). No mutations in this panel were detected in the remaining 30 samples. A total of 63 HR gene mutations were detected. *ATM* (16/484, 3.31%), *ATRX* (14/484, 2.89%), and *ATR* (13/484, 2.69%) were among the most frequently mutated HR genes, whereas no alterations in *CHEK2* were detected in the Chinese cohort (**Figure 1A**). Fifty-one of the 484 (10.54%) patients had at least one somatic mutation in an HR gene. The mutational frequencies of HR genes in the Chinese cohort were lower than those in the cohort from TCGA (**Supplementary Figure S1B**).

Germline mutations in HR genes, particularly *PALB2*, *BRCA1*, and *RAD51C*, have been reported to increase GC risk^27^. We next investigated germline HRD in these 484 patients. Pathogenic germline variants were identified in 15 of the 484 (3.10%) patients, although no participants carried both germline and somatic HR gene mutations (**Figure 1B**). The 15 pathogenic mutations occurred in 6 genes: *ATM* (6/15, 40.00%), *RAD51C* (2/15, 13.33%), *BRCA1* (2/15, 13.33%), *BRCA2* (2/15, 13.33%), *CHEK2* (2/15, 13.33%), and *PALB2* (1/15, 6.67%) (**Figure 1C**). The details of all deleterious germline mutations are shown in **Supplementary Table S2**. *ATM* (somatic or germline) was the most frequently mutated gene, but only 4 patients in our cohort carried germline *BRCA1/2* mutations (one patient had both germline *BRCA2* and somatic *ATM* mutations).

Although HRD was initially defined by the presence of germline mutations in HR genes, clinical evidence has shown that somatic mutations in HR genes are associated with sensitivity to poly-ADP ribose polymerase inhibitors (PARPi)^28–30^. Thus, participants with these mutations were included in the HR-mut group (*n =* 51) in the subsequent analyses.

### Alterations in genetic interactions between the HR-mut and HR-wt groups

To better understand how HRD alters genomic patterns in GC, we compared the mutation frequencies between the HR-mut and HR-wt patient groups. Of the 32 genes with significant differences, only *TP53* tended to be mutated in the HR-wt group (**Figure 1D**). In addition, alterations in genes associated with PI3K-ERBB signaling, including *MTOR*,* PIK3R1*, *ERBB2*, and* ERBB4*, occurred more frequently in the HR-mut group (**Figure 1D**). We further explored cooccurring mutations in the HR-mut group (all 63 somatic mutated HR genes were categorized as HR-mut) and found co-occurrence of mutations in *KRAS/NTRK3/FBXW7/ERBB4/SOX9/FAT1/ARID1B/NF1/PREX2/SMARCA4/KMT2C*; somatic *TP53* and somatic HR gene mutations were mutually exclusive (all *P* < 0.05) (**Figure 1E**).

### Mutations in HR genes indicate a high TMB

**Figure 2:**
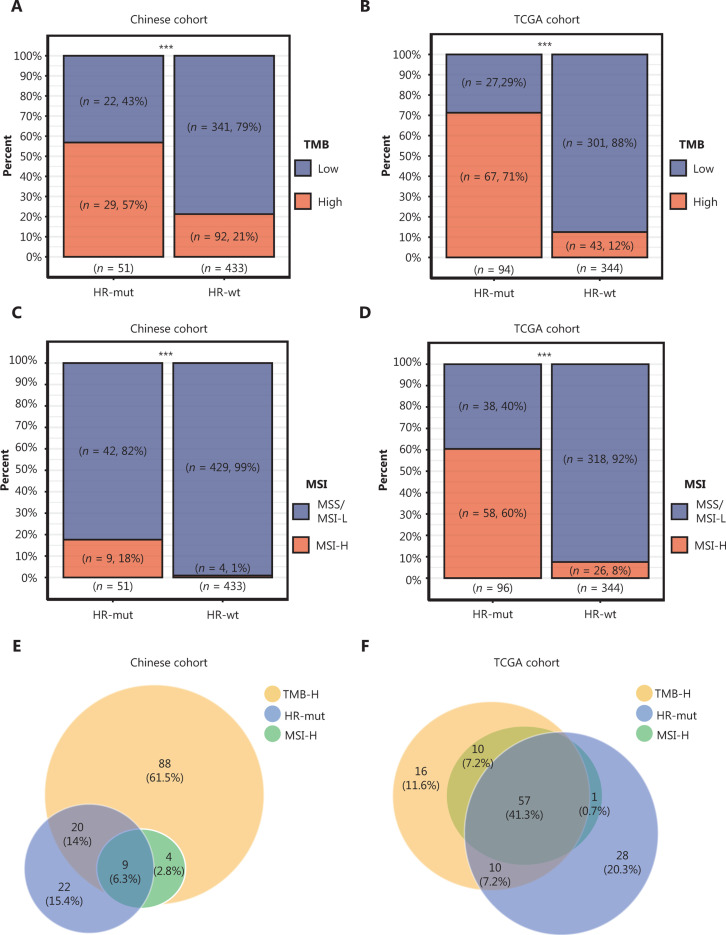
Associations among mutations in HR genes, tumor mutation burden (TMB) and microsatellite instability (MSI) status. (A, B) Bar plots display the percentages of TMB-high patients in the HR-mut group compared with the HR-wt group in the Chinese (A) and TCGA (B) cohorts. (C, D) Bar plots display the percentage of MSI-H patients in the HR-mut group compared with the HR-wt group in the Chinese (C) and TCGA (D) cohorts. Comparisons between groups were performed with Fisher’s exact test (***P < 0.001). (E, F) Venn diagrams illustrating the overlap among HR-mut, TMB-H, and MSI-H patients in the Chinese (E) and TCGA (F) cohorts.

Recently, a phase Ib/II clinical trial (NCT02915432) showed that in GC, patients with a high TMB respond significantly better to anti-PD-1 antibody (toripalimab, JS001) therapy than patients with low TMB^31^. We next investigated the correlation between HRD and the TMB and found a significantly higher TMB in HR-mut GC than in HR-wt GC (the 75th percentile of the TMB value was defined as TMB-H; see “Materials and methods”; Fisher’s exact test, *P* < 0.001; **Figure 2A**). Because loss of *BRCA1/2* can confer an HRD phenotype in various tumor types^6,32^, we also assessed molecular features in the* BRCA1/2*-mut group. In the Chinese cohort, a higher proportion of patients with high TMB was observed in the somatic *BRCA1/2-*mut group than the *BRCA1/2*-wt group (Fisher’s exact test, *P* < 0.01; **Supplementary Figure S2A**). To validate the positive correlation between HRD and the TMB, we next analyzed the 438 TCGA patient cohort diagnosed with GC. According to these data, both HR-mut (*n =* 94) and *BRCA1/2*-mut (*n =* 37) groups comprised a larger fraction of patients with a high TMB than the corresponding wt groups (all *P* < 0.001; **Figure 2B**, **Supplementary Figure S2B**). These results demonstrate that somatic mutations in HR genes correlate significantly and positively with the TMB in GC.

### Mutations in HR genes are associated with MSI status

On the basis of the results of the KEYNOTE-059 trial (NCT02335411), MSI may be a biomarker predictive of the response to pembrolizumab^33^. Herein, we classified samples as MSI-H or MSS/MSI-L (see “Materials and Methods”). In the Chinese cohort, patients with MSI-H genomic signatures were significantly enriched in HR-mut [18% *vs.* 1% (MSI-L/MSS), *P* < 0.001; **Figure 2C**] and *BRCA1/2-*mut (33% *vs.* 2%, *P* < 0.01; **Supplementary Figure S2C**). Similar results were obtained for the cohort from TCGA (**Figure 2D**, **Supplementary Figure S2D**). Venn diagrams depicted the relationships among TMB-H, MSI-H, and HR-mut/*BRCA1/2-*mut in GC in both our cohort (**Figure 2E**) and that from TCGA (**Figure 2F**). Moreover, almost all MSI-H cases had a high TMB, in agreement with findings from a previous study from Foundation Medicine^34^.

### Mutations in HR genes are associated with tumor aneuploidy and tumor immunogenicity

**Figure 3:**
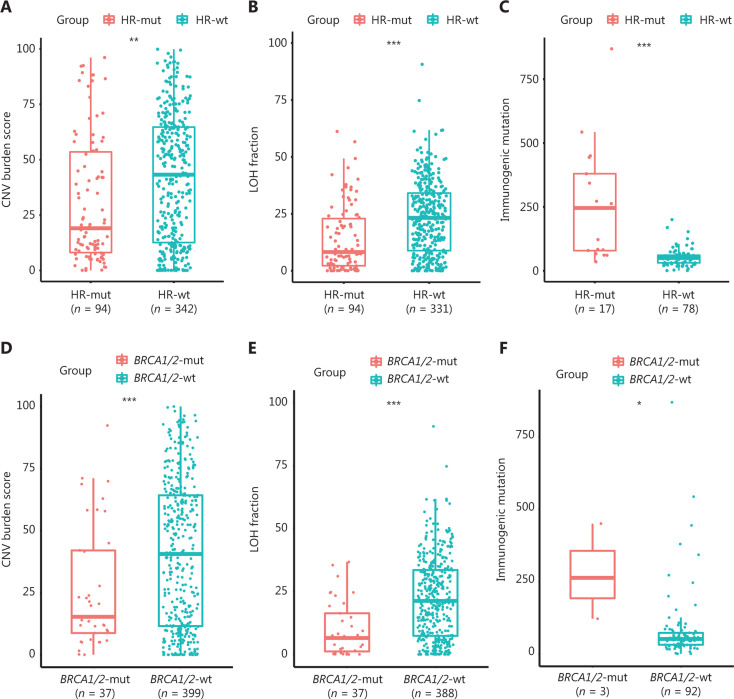
Mutations in HR genes are associated with tumor aneuploidy and tumor immunogenicity. (A, D) HR-mut GC (A) and *BRCA1/2*-mut GC (D) had significantly lower copy number variation burden scores than the corresponding wt groups. (B, E) The LOH fraction is significantly smaller in HR-mut GC (B) and *BRCA1/2*-mut GC (E) than in the corresponding wt groups. (C, F) Immunogenic mutations are significantly higher in HR-mut GC (C) and *BRCA1/2*-mut GC (F) than in the corresponding wt groups. *P*-values were calculated with the Wilcoxon test; the box shows the upper and lower quartiles (*P < 0.05, **P < 0.01, ***P < 0.001).

We next evaluated tumor aneuploidy, which correlates negatively with the immunotherapeutic response^21^ in the cohort from TCGA. Interestingly, somatic copy number alterations (SCNAs) and LOH levels were markedly lower in HR-mut GC (Wilcoxon test,* P* < 0.01; **Figure 3A**; *P* < 0.001; **Figure 3B**). Furthermore, HR-mut GC exhibited significantly more immunogenic mutations (gene sets in **Supplementary Table S3**) than HR-wt GC (*P* < 0.001; **Figure 3C**). Similar results were found for *BRCA1/2*-mut GC (Wilcoxon test,* P* < 0.001; **Figure 3D**; *P* < 0.001; **Figure 3E**; *P* < 0.05; **Figure 3F**). Thus, the HRD phenotype (conferred by either HR-mut or *BRCA1/2*-mut status) may influence immune evasion and can be used to predict the efficacy of ICIs.

### HR-mut status is associated with elevated immune activity and a favorable ICI therapeutic response

**Figure 4:**
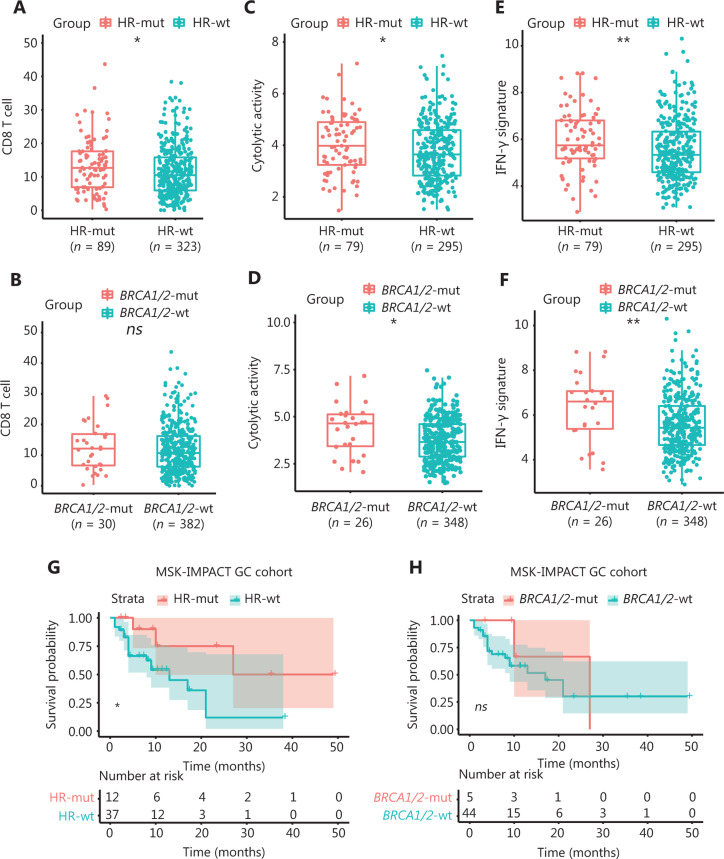
HR-mut status is associated with elevated immune activity and a favorable ICI therapeutic response. (A) The fraction of CD8-positive T cells, as inferred with CIBERSORT, is higher in HR-mut GC than in HR-wt GC. (B) The fraction of CD8-positive T cells shows no significant difference in *BRCA1/2*-mut GC compared with *BRCA1/2*-wt GC. (C, D) Cytolytic activity is significantly higher in HR-mut GC (C) and *BRCA1/2*-mut GC (D) than in the corresponding wt groups. (E, F) The IFN-γ signature is significantly higher in HR-mut GC (E) and *BRCA1/2*-mut GC (F) than in the corresponding wt groups. *P*-values were calculated with the Wilcoxon test; the box shows the upper and lower quartiles. (G, H) Kaplan–Meier survival curves show the overall survival (OS) stratified by HR-mut/wt (G) and *BRCA1/2*-mut/wt (H) in the MSK-IMPACT GC cohort. *P*-values were obtained with the log-rank test (*P < 0.05; **P < 0.01; ns, P > 0.05).

A recent study has reported elevated levels of TILs in HRD ovarian carcinoma^5^. To further determine whether such an increase might exist in GC, we downloaded TCGA immune subpopulation profiles, as calculated by a deconvolution approach (CIBERSORT^23^; see “Materials and methods”). HR-mut GC showed significantly higher abundance of CD8+ T cells, follicular helper CD4 T (T_FH_) cells, M1 macrophages, activated mast cells and activated memory CD4+ T cells than HR-wt GC (Wilcoxon test, *P* < 0.05; **Figure 4A**; **Supplementary Figure S3A**). However, only M1 macrophages and activated memory CD4+ T cells were enriched in *BRCA1/2*-mut GC, as compared with *BRCA1/2*-wt GC (**Figure 4B**, **Supplementary Figure S3B**) (Wilcoxon test, *P* < 0.01).

Next, we analyzed whether immune-related signatures correlating with the ICI response were altered in HRD GC by analyzing the IFN-γ-responsive signature (gene sets in **Supplementary Table S4**)^25^ and the cytolytic activity signature^24^ in gene expression profiles by using RNA data from the GDC Data Portal (https://portal.gdc.cancer.gov/; see “Materials and Methods”). Interestingly, the levels of these 2 immune signatures were higher in HR-mut and *BRCA1/2*-mut GC than in HR-wt and* BRCA1/2*-wt GC (**Figure 4C–4F**). Moreover, the expression of immune checkpoint-related molecules (*CD274* and* PDCD1*) was also significantly up-regulated in the HR-mut group (**Supplementary Figure S4**).

In contrast, Epstein–Barr virus (EBV)-associated GC (EBVaGC) has been reported to display elevated tumor-infiltrating T-cell subsets^35^. Hence, we further investigated the relationship between EBV infection and immune characteristics in the TCGA cohort. Overall, EBV-positive status was associated with more intense CD4+ memory T and CD8+ T cell infiltration (**Supplementary Figure S5A and 5B**), a finding consistent with results from previous research^36^. In addition, we found that the immune-related signatures correlating with the ICI response (cytolytic activity signature and IFN-γ signature) were also enriched in EBVaGC (**Supplementary Figure S5C and 5D**). In the following analysis, we found only 3 patients with HR mutation EBV infection, thus suggesting that HR mutation and EBV infection may be mutually exclusive. In addition, a further stratified analysis showed that, compared with the HR-wt/EBV-neg and HR-mut/EBV-pos groups, the HR-wt/EBV-pos group showed the greatest immune activity (**Supplementary Figure S5E–5H**), thus potentially explaining why EBV infected patients respond to immunotherapy^37^. In the EBV negative group, HR mut patients exhibited higher CD8+ T cell infiltration, a cytolytic activity signature and an IFN-γ signature, thereby indicating that HR mutation can further identify EBV negative patients who might benefit from ICIs. Finally, we explored the correlation between HRD and the ICI response by using an ICI-treated cohort (MSK-IMPACT cohort)^26^. A total of 49 patients with STAD or GEJ were enrolled in the MSK-IMPACT cohort. The HR-mut GC (*n =* 12) group had a significantly better OS than the HR-wt GC group (*n =* 37) (log-rank test,* P* < 0.05; Figure 4G). However, the correlation between the mutation status and OS was nonsignificant in BRCA1/2-mut GC (log-rank test, P > 0.05; **Figure 4H**). As mentioned in the original literature, most patients (94% of tumors excluding glioma) in the MSK-IMPACT cohort had stage IV or metastatic disease, and the OS was measured from the date of first ICI treatment to the time of death or most recent follow-up^26^. Although the following conclusion is not sufficient, we propose that the OS was mainly a benefit of the ICI treatment.

These results suggest that HR-mut status is associated with elevated immune activity in GC and that HR-mut status might be a better marker of the ICI response than somatic mutations in *BRCA1*/2. Thus, detection of somatic mutations in HR genes might aid in identifying patients who might benefit from immune checkpoint blockade therapy.

## Discussion

**Figure 5:**
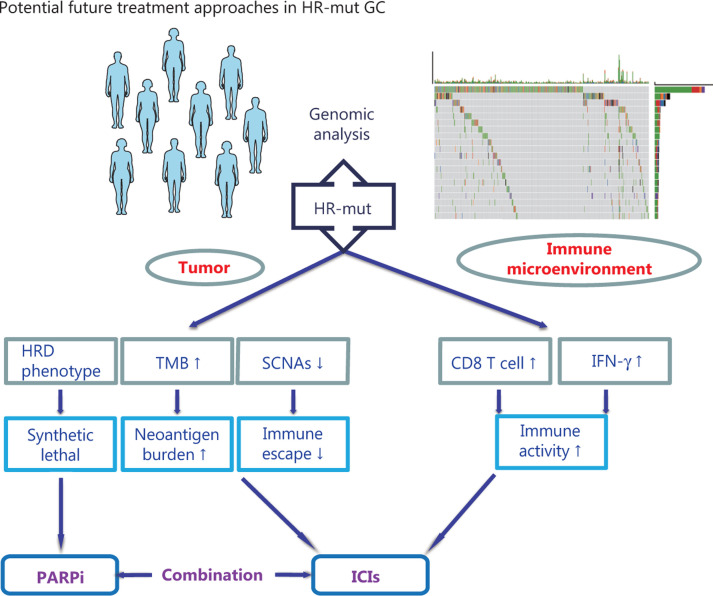
Potential future treatment approaches in HR-mut GC. Homologous recombination deficiency (HRD); tumor mutational burden (TMB); somatic copy number alterations (SCNAs); interferon-gamma (IFN-γ); poly-ADP ribose polymerase inhibitors (PARPi); immune checkpoint inhibitors (ICIs).

This study examined the mutational landscape of HR genes in Chinese patients with GC. The results indicate that 10.54% (**Figure 1A and 1B**) of Chinese patients with GC may benefit from various treatments associated with the HR pathway (**Figure 5**).

First, HRD is a well-known predictive biomarker for the response to PARPi. PARPi induce synthetic lethal effects in cancer cells lacking functional HR^38^ and are currently approved for the treatment of ovarian and breast cancer with *BRCA1/2* mutations^39–41^. However, according to our data, the total percentage (2.69%, 13/484) of nonsilent somatic and pathological germline mutations in *BRCA1/2* in GC was not as high as that in gynecological cancers^42,43^. Moreover, the GOLD study (NCT01924533) did not meet its primary endpoint in the ATM-negative group^44^. Therefore, exploring other biomarkers of HRD to predict the response to PARPi in GC is necessary.

In this study, we defined 2 groups (HR-mut and* BRCA1/2-*mut) that might have an HRD phenotype. Our analysis revealed that HRD (conferred by HR-mut) was significantly associated with genetic features associated with the immunotherapeutic response in GC. Greater TMB and MSI correlated positively with HR-mut status in the Chinese cohort, and this finding was verified in the cohort from TCGA (**Figure 2**). Both an increased mutational load and MSI-H status have been correlated with ICI response in various trials^45^. However, the relationship between HRD and the ICI response is clearly more complex than previously believed. Tumor aneuploidy (or the SCNA level) is a newly discovered immune tolerance mechanism that correlates with markers of immune evasion^21^. Previous research has shown that the combination of the tumor SCNA level and the TMB is a better predictor of survival after immunotherapy than either biomarker alone^21^. Thus, the increased TMB and decreased SCNA levels in HR-mut GC suggest an improved immunotherapeutic response in this type of cancer. In addition, to our knowledge, the negative correlation between the SCNA level and HR-mut status that we found in GC has not been previously reported.

In addition, the status of TILs, particularly CD8+ T cells, has been verified as the core determinant of ICI treatment efficacy^46,47^. Therefore, the increased CD8+ T cell infiltration in HR-mut GC observed in this study further demonstrates that somatic mutations in HR genes are strongly associated with elevated immune activity. Indeed, we found that antitumor immune signatures increased in HR-mut GC and that patients with HR-mut GC had significantly better OS than those with HR-wt GC after ICI treatment (**Figure 4**). Although the *BRCA1/2-*mut group did not show the exact same trends as the HR-mut group, it did exhibit an increasing trend in immune activity. In contrast, these data indicate that HR-mut status may be a better marker of the response to ICIs than somatic mutations in *BRCA1*/2.

Interestingly, EBV infection and HR-mut status appear to be mutually exclusive, although most patients with GC with MSI-H are HR-gene mutation carriers. A previous study has proposed a molecular classification dividing gastric cancer into 4 subtypes—tumors positive for EBV, microsatellite unstable tumors, genomically stable tumors, and tumors with chromosomal instability—thus implying that EBV infected patients usually belong to the microsatellite stable group. Given that HR-mut patients tend to be microsatellite unstable, mutual exclusivity of HR-mut and EBV infection seems reasonable^48^. Although we cannot explain this observation, this result implies that HR-mut GC is a subtype with special molecular characteristics.

GC is highly heterogeneous, thus making the practical application of molecular-targeted drugs difficult^3^. Nevertheless, the heterogeneity of GC suggests that patients with advanced GC can derive more benefit from combination therapies than from monotherapies. Widespread clinical studies combining PARPi and ICIs are currently being conducted^49^. On the basis of our results, HRD is a potential biomarker of the response to both PARPi and ICIs, thus indicating that HRD GC may respond to combination therapy with PARPi/ICIs. Furthermore, we observed that alterations associated with PI3K-ERBB signaling occurred more frequently in HR-mut GC than in HR-wt GC (**Figure 1D**). This pathway is being targeted in several ongoing trials, including NCT00879333 (everolimus, RAD001), NCT01613950 (alpelisib, BYL719), and NCT01896531 (ipatasertib, GDC-0068) (www.clinicaltrials.gov). Our data suggest that the mutational profile of HR genes can serve as a stratification factor in such trials.

This study has several limitations. First, owing to data restrictions, we could not evaluate HLA LOH alteration, which is a better immune escape marker than is the LOH fraction. Second, we lacked transcriptome data for the Chinese cohort to validate the findings obtained from TGCA. Therefore, further studies are warranted.

## Conclusions

In summary, our data suggest that detection of somatic mutations in HR genes might aid in identifying patients who might benefit from immune checkpoint blockade therapy. In addition, the molecular features of HRD GC provide new opportunities to predict the tumor response to multiple treatments.
