# Supplementary material for: The mutational pattern of homologous recombination (HR)-associated genes and its relevance to the immunotherapeutic response in gastric cancer
Source: Cancer Biol Med. 2020 Dec 15;17(4):1002–13. doi: 10.20892/j.issn.2095-3941.2020.0089 (PMC7721103; doi:10.20892/j.issn.2095-3941.2020.0089)
Supplement: Supplementary file 1 [file cbm-17-1002-s001.pdf]

Supplementary materials

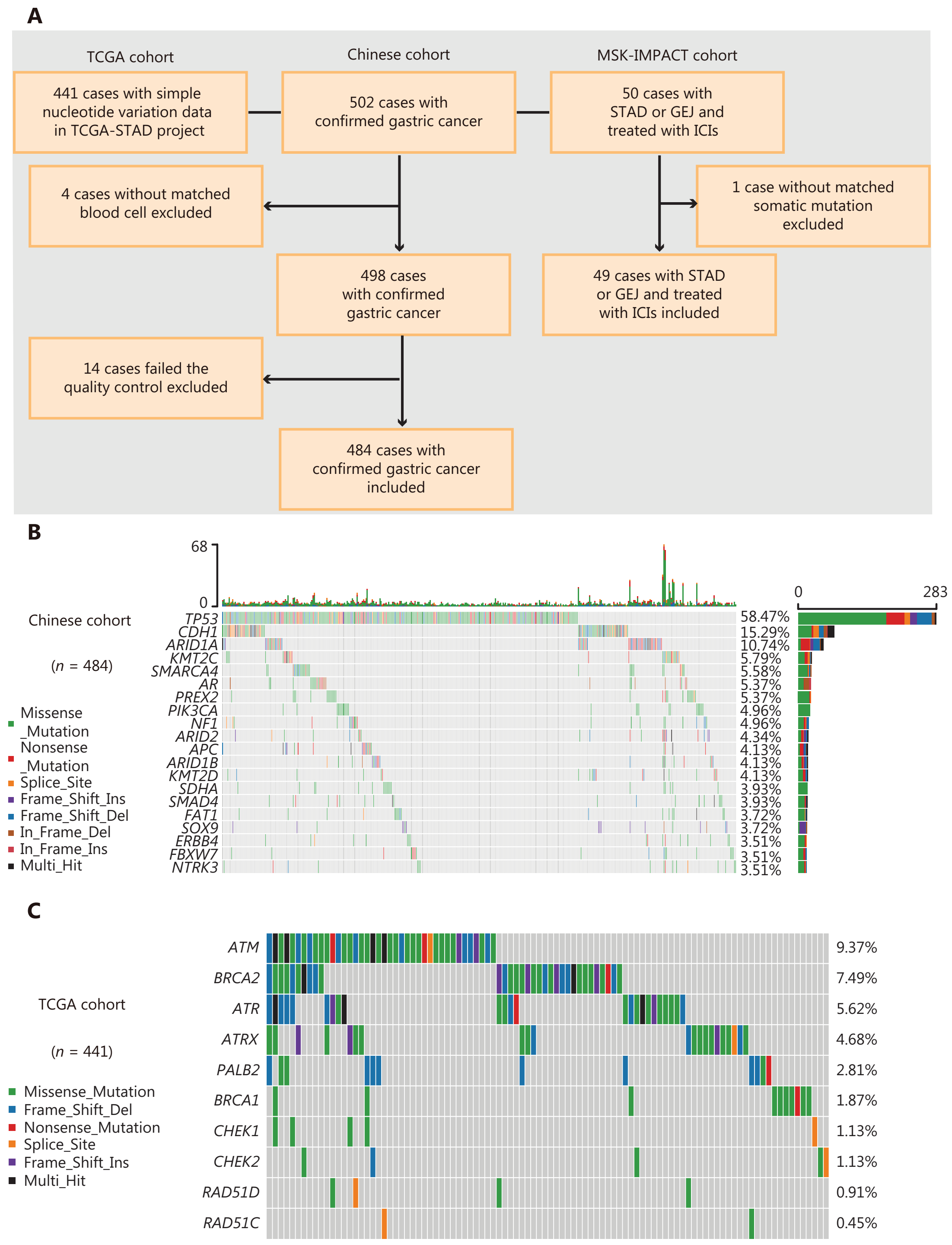

Figure S1 Landscape of GC patient mutations. (A–C) Flow chart for participant selection (A), mutational landscape of the top 20 mutated genes in the Chinese cohort (B), and HR genes in the cohort from TCGA (C). The columns and rows represent patients and genes, respectively, and are sorted by the number of patients in which a gene is mutated, in descending order. The right panel indicates the frequency of gene mutations. Mutation types are differentiated by colors. Thirty samples with no mutations detected from this panel are not shown in this figure.

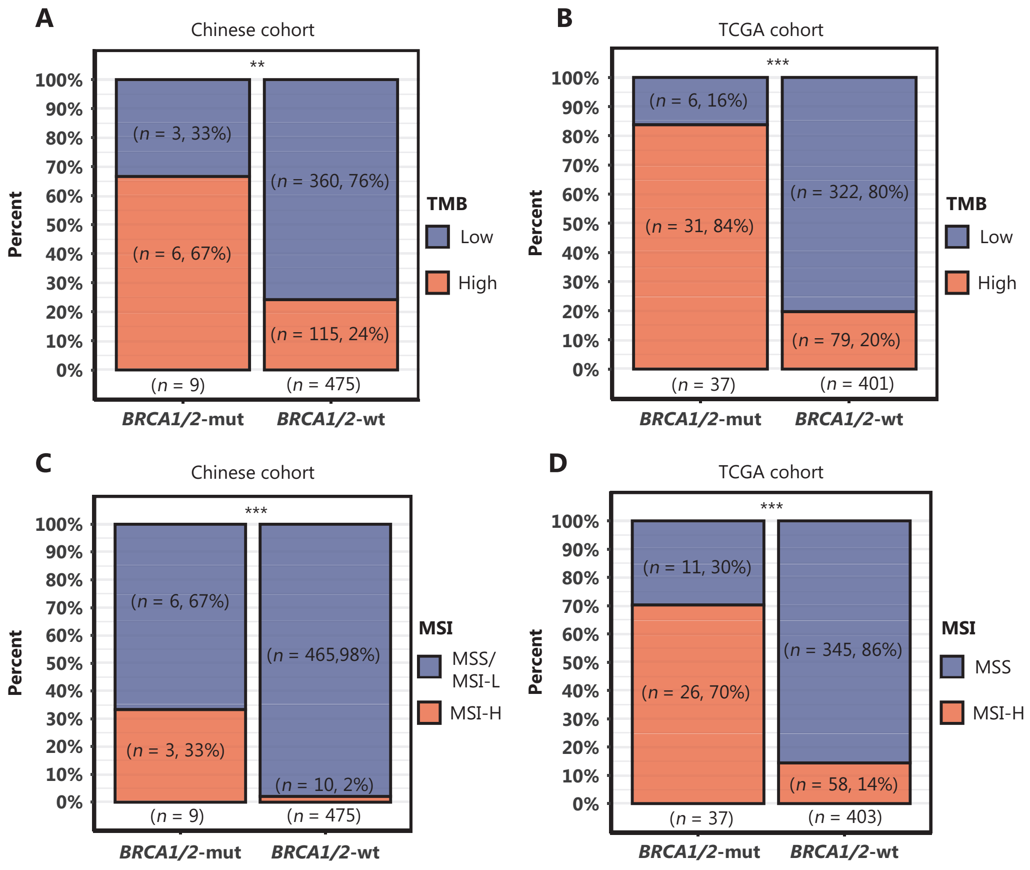

Figure S2 Associations among mutations of *BRCA1/2*-mut, the tumor mutation burden (TMB) and the microsatellite instability (MSI) status. (A, B) Bar plots display the percentage of TMB-high patients in the *BRCA1/2*-mut group compared with the *BRCA1/2*-wt group in the Chinese (A) and TCGA (B) cohorts. (C, D) Bar plots display the percentage of MSI-H patients in the *BRCA1/2*-mut group compared with the *BRCA1/2*-wt group in the Chinese (C) and TCGA (D) cohorts. Comparisons between groups were performed with Fisher's exact test (**$P < 0.01$, ***$P < 0.001$).

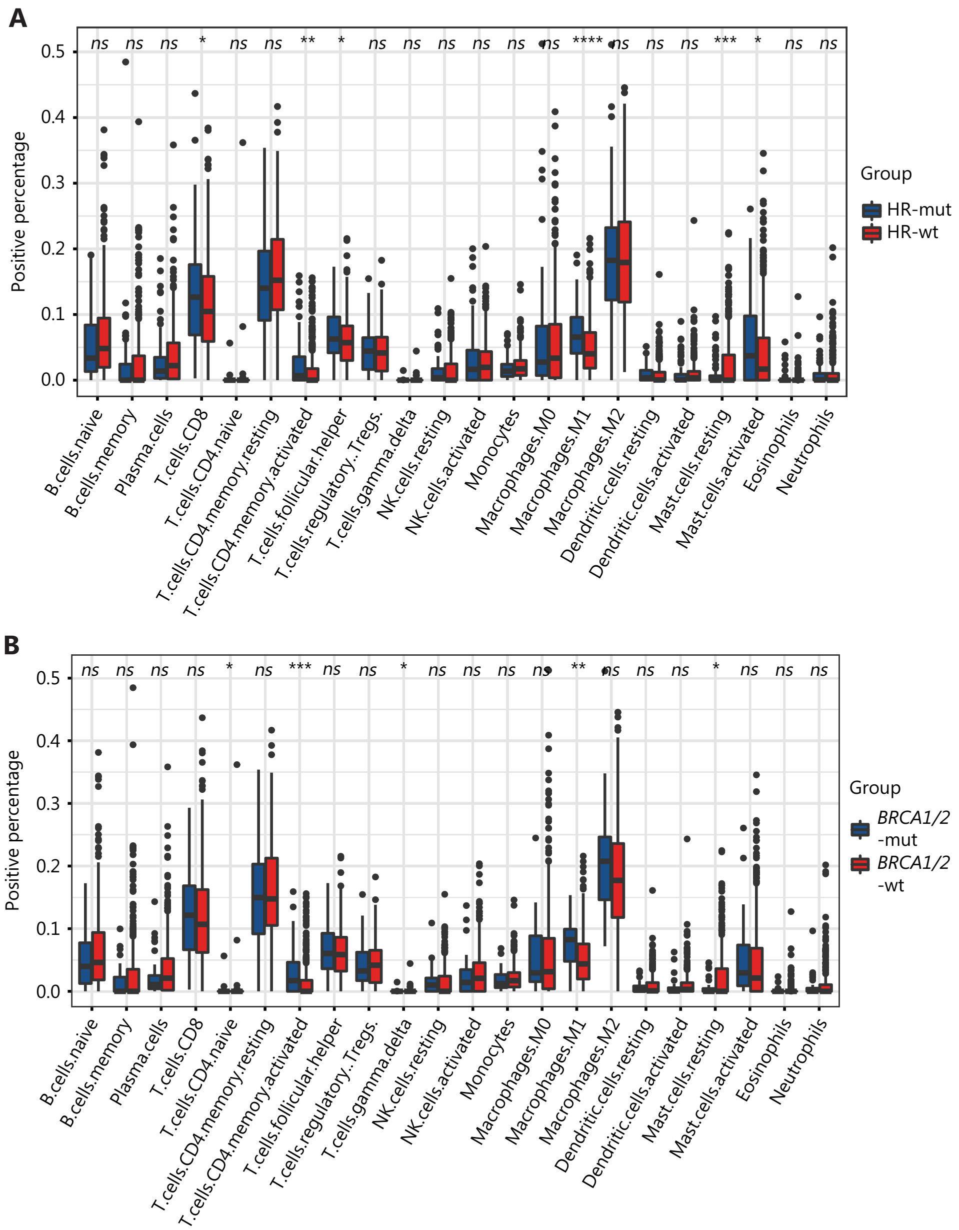

Figure S3 Fraction of leukocyte type content, as inferred with CIBERSORT in GC. (A, B) Estimated leukocyte proportions in HR-mut GC (A) and *BRCA1/2*-mut GC (B) compared with the corresponding wt groups. *P*-values were calculated with the Wilcoxon test; the box shows the upper and lower quartiles (**P* < 0.05; ***P* < 0.01; ****P* < 0.001; *****P* < 0.0001; ns, *P* > 0.05).

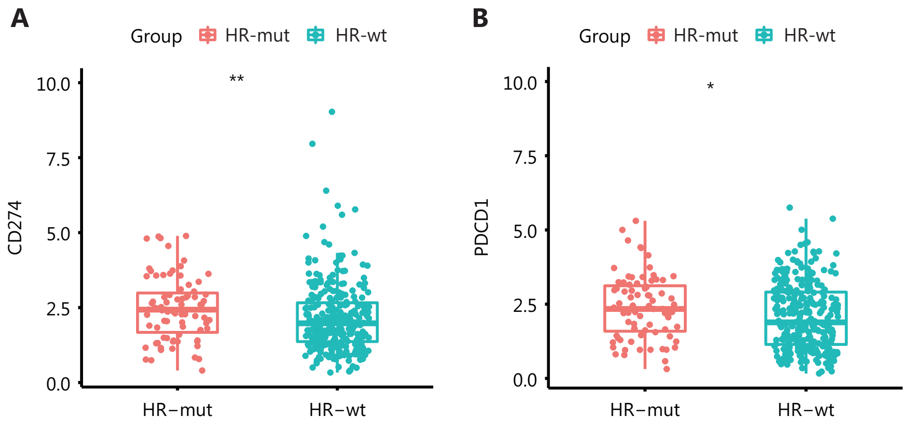

Figure S4 HR-mut status is associated with elevated expression level of immune checkpoint molecules. (A, B) Expression of *CD274* (A) and *PDCD1* (B) in HR-mut GC is greater than that in the HR-wt group. *P*-values were calculated with the Wilcoxon test; the box shows the upper and lower quartiles (*P < 0.05; **P < 0.01).

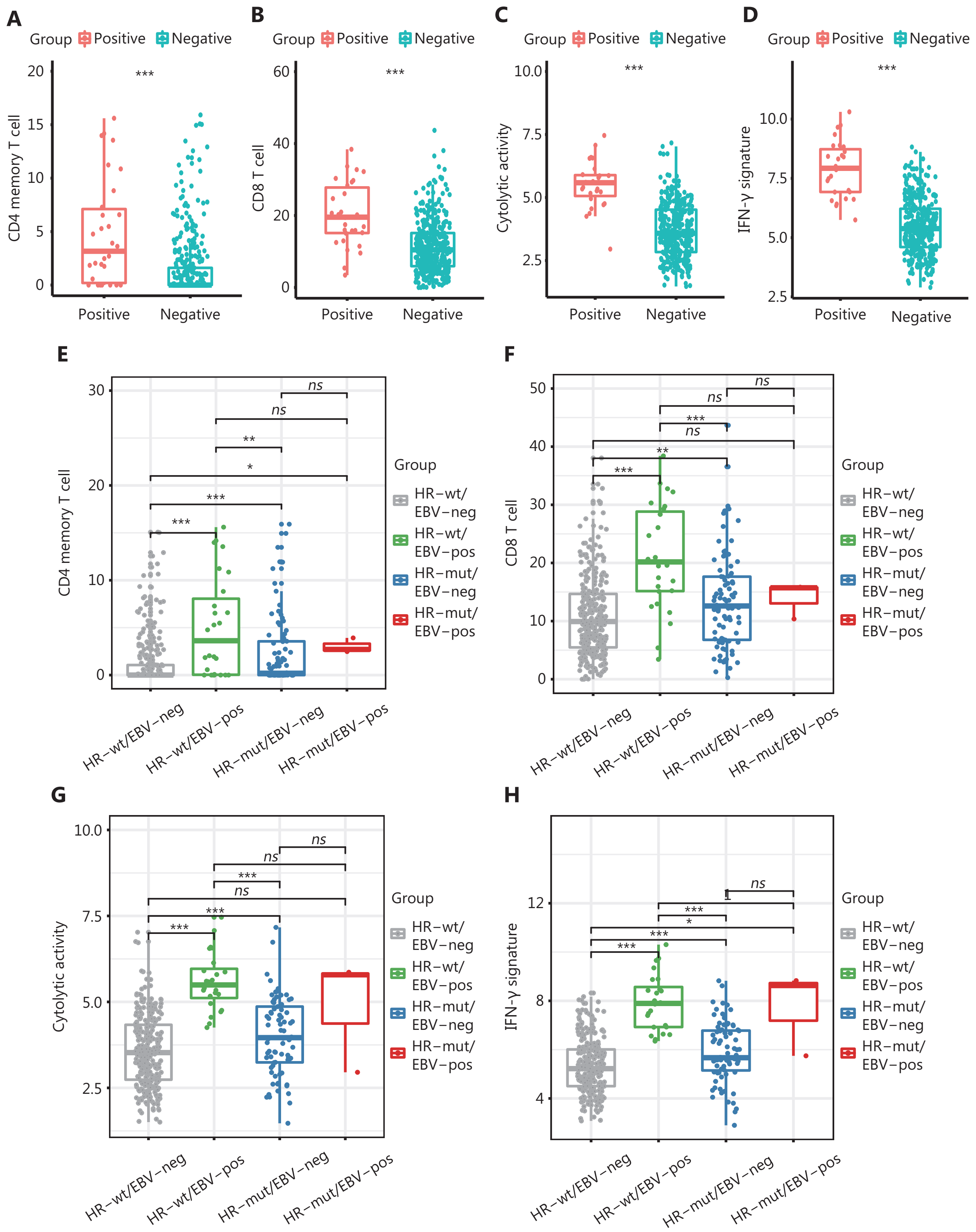

Figure S5 Associations among EBV infection status, HR-mut status and immune activity in GC. (A) The fraction of CD4 memory T cells, as inferred with CIBERSORT, is higher in EBV positive GC than in EBV negative GC. (B) The fraction of CD8-positive T cells is higher in EBV positive GC than in EBV negative GC. (C, D) Cytolytic activity (C) and IFN-γ signature (D) is significantly higher in EBV positive GC than in EBV negative GC. (E-H) Box plots show the fraction of CD4 memory T cells (E), fraction of CD8-positive T cells (F), cytolytic activity (G) and IFN-γ signature (H) in the HR-wt/EBV-neg, HR-wt/EBV-pos, HR-mut/EBV-neg, and HR-mut/EBV-pos groups. *P*-values were calculated with the Wilcoxon test; the box shows the upper and lower quartiles (**P* < 0.05; ***P* < 0.01; ****P* < 0.001; ns, *P* > 0.05).

Table S1 Demographic distribution of the Chinese cohort (n = 484), TCGA cohort (n = 441), and MSK-IMPACK cohort (n = 49)

Characteristics	Chinese cohort		TCGA cohort		MSK-IMPACK cohort	
	n (%)	Mean (SD)	*n* (%)	Mean (SD)	*n* (%)	Mean (SD)
Age		57.39 (13.04)		65.69 (10.74)		61.12 (13.33)
Sex						
Male	312 (64.5)		283 (64.2)		37 (75.5)	
Female	172 (35.3)		158 (35.8)		12 (24.5)	
Stage						
I	11 (2.3)		59 (13.4)			
II	7 (1.4)		130 (29.5)			
III	177 (36.6)		186 (42.2)			
IV	278 (57.4)		43 (9.8)			
Missing data	11 (2.3)		23 (5.2)			
EBV						
Positive			30 (6.8)			
Negative			353 (80.0)			
Missing data			58 (13.2)			

Table S2 Details of all deleterious germline mutations in HR genes in the Chinese cohort

Sample_id	Gene_refGene	Chrom	Depth	Mutation_g	Mutation_c	Mutation_p
P176898_STAD	ATM	chr11	612	chr11:g.108128241_108128242delCT	c.2284_2285delCT	p.L762Vfs*2
P178947_STAD	ATM	chr11	380	chr11:g.108160488C>T	c.4396C>T	p.R1466*
P179015_STAD	ATM	chr11	503	chr11:g.108121594_108121595delAA	c.1402_1403delAA	p.K468Efs*18
P186259_STAD	ATM	chr11	167	chr11:g.108115727C>T	c.875C>T	p.P292L
P186371_STAD	ATM	chr11	347	chr11:g.108178646C>A	c.5697C>A	p.C1899*
P193132_STAD	ATM	chr11	492	chr11:g.108196887delG	c.6910delG	p.E2304Sfs*6
P191191_STAD	BRCA1	chr17	385	chr17:g.41201209delG	c.5335delC	p.Q1779Nfs*14
P186915_STAD	BRCA1	chr17	187	chr17:g.41276044A>G	c.70T>C	p.C24R
P186190_STAD	BRCA2	chr13	288	chr13:g.32911601C>T	c.3109C>T	p.Q1037*
P177494_STAD	BRCA2	chr13	345	chr13:g.32911542T>G	c.3050T>G	p.I1017S
P189032_STAD	CHEK2	chr22	188	chr22:g.29083962G>A	c.1555C>T	p.R519*
P189606_STAD	CHEK2	chr22	436	chr22:g.29121258G>T	c.417C>A	p.Y139*
P181878_STAD	PALB2	chr16	450	chr16:g.23637612C>T	c.2693G>A	p.W898*
P184904_STAD	RAD51C	chr17	347	chr17:g.56787223C>T	c.709C>T	p.R237*
P193918_STAD	RAD51C	chr17	440	chr17:g.56772540dupA	c.394dupA	p.T132Nfs*23

Table S3 Immune signature gene sets

pDCs	Co-inhibition, APC	Type II IFN response	Cytolytic activity	MHC class I	CD8+ T cells	Co-inhibition, T cell	Co-stimulation, APC
LILRA4	PDCD1LG2	GPR146	GZMA	HLA-A	CD8A	LAG3	ICOSLG
CLEC4C	CD274	SELP	PRF1	B2M		CTLA4	CD70
PLD4	C10orf54	AHR		TAP1		CD274	TNFSF14
PHEX	LGALS9					CD160	CD40
IL3RA	PVRL3					BTLA	TNFSF9
PTCRA						C10orf54	TNFSF4
IRF8						LAIR1	TNFSF15
IRF7						HAVCR2	TNFSF18
GZMB						CD244	TNFSF8
CXCR3						TIGIT	SLAMF1
							CD58

pDCs, plasmacytoid dendritic cells; APC, adenomatous polyposis coli; IFN, interferon; CD8+, CD8-positive.

Table S4 IFN-γ signature gene sets

Genes
IDO1
CXCL10
CXCL9
HLA-DRA
STAT1
IFNG
